# Surgery/Anesthesia disturbs mitochondrial fission/fusion dynamics in the brain of aged mice with postoperative delirium

**DOI:** 10.18632/aging.102659

**Published:** 2020-01-12

**Authors:** Yayuan Lu, Lei Chen, Jishi Ye, Chang Chen, Ying Zhou, Ke Li, Zongze Zhang, Mian Peng

**Affiliations:** 1 Department of Anesthesiology, Zhongnan Hospital of Wuhan University, Wuhan, China; 2 Department of Anesthesiology, Renmin Hospital of Wuhan University, Wuhan, China

**Keywords:** postoperative delirium, mitochondrial dynamics, mitochondrial function, hippocampus, prefrontal cortex

## Abstract

Postoperative delirium (POD) is a common complication following surgery and anesthesia (Surgery/Anesthesia). Mitochondrial dysfunction, which is demonstrated by energy deficits and excessively activated oxidative stress, has been reported to contribute to POD. The dynamic balance between mitochondrial fusion and fission processes is critical in regulating mitochondrial function. However, the impact of Surgery/Anesthesia on mitochondrial fusion/fission dynamics remains unclear. Here, we evaluate the effects of laparotomy under 1.4% isoflurane anesthesia for 2 hours on mitochondrial fission/fusion dynamics in the brain of aged mice. Mice in Surgery/Anesthesia group showed unbalanced fission/fusion dynamics, with decreased DISC1 expression and increased expression of Drp1 and Mfn2 in the mitochondrial fraction, leading to excessive mitochondrial fission and disturbed mitochondrial morphogenesis in the hippocampus and prefrontal cortex. In addition, surgical mice presented mitochondrial dysfunction, demonstrated by abnormally activated oxidative stress (increased ROS level, decreased SOD level) and energy deficits (decreased levels of ATP and MMP). Surgery/Anesthesia also decreased the expression of neuronal/synaptic plasticity-related proteins such as PSD-95 and BDNF. Furthermore, Surgery/Anesthesia induced delirium-like behavior in aged mice. In conclusion, Surgery/Anesthesia disturbed mitochondrial fission/fusion dynamics and then impaired mitochondrial function in the brain of aged mice; these effects may be involved in the underlying mechanism of POD.

## INTRODUCTION

Delirium is an acute, transient, usually reversible, fluctuating disturbance in attention, cognition, and consciousness level [1]. Postoperative delirium (POD) is a common complication following surgery and anesthesia, especially in the elderly population [2–4]. POD may lead to greater lengths of hospital stay, increased hospitalization costs, decreased life independence, and increased morbidity and mortality; furthermore, it has the potential to induce long-term cognitive dysfunction and even dementia [2, 5–8]. Advanced age was reported to be an independent risk factor for the development of POD [9], as the elderly may have a lack of physiologic reserves [10, 11]. With a significant increase in the aging population, complications specifically related to the elderly are becoming increasingly important [12].

Mitochondrial dysfunction, which is demonstrated by energy deficits and excessively activated oxidative stress, has been reported to contribute to POD [13]. Mitochondria are dynamic organelles that continually move, fuse, and divide [14]. Mitochondrial dynamics include mitochondrial biogenesis, selective degradation (including mitophagy), mitochondrial fusion and fission events, as well as processes such as intracellular transport [15, 16]. Furthermore, the dynamic balance between mitochondrial fusion and fission processes is critical in regulating mitochondrial function [15]. Research showed that mitochondrial fission/fusion dynamics play an important physiological role in the development of the nervous system and synaptic plasticity via regulation of mitochondrial functions, which include adenosine triphosphate (ATP) production, Ca^2+^ buffering, neurotransmitter synthesis and degradation, reactive oxygen species (ROS) production and sequestration, apoptosis, and intermediate metabolism [17]. However, the impact of surgery and anesthesia on mitochondrial fusion and fission dynamics largely remain to be determined.

Therefore, we hypothesized that Surgery/Anesthesia may disturb mitochondrial fission/fusion dynamics and then impair mitochondrial function in the brain of aged mice, thus resulting in the occurrence and development of POD. In this study, we aimed to evaluate the effects of surgery (laparotomy) under 1.4% isoflurane anesthesia (Surgery/Anesthesia) for 2 hours on mitochondrial fission/fusion dynamics in the brain of aged mice by evaluating the mitochondrial morphometrics in the hippocampus and prefrontal cortex, expression of the mitochondrial fission/fusion dynamics-related proteins (disrupted in schizophrenia 1 [DISC1], dynamin-related protein 1 [Drp1], and mitofusin 2 [Mfn2]), and identifying the subsequent changes in mitochondrial function-related indicators, namely, oxidative stress and anti-oxidative stress-related markers (ROS and superoxide dismutase [SOD]), energy metabolism-related indicators (ATP and mitochondrial membrane potential [MMP]), and neuronal/synaptic plasticity-related proteins. The findings of this investigation may be helpful in identifying new underlying mechanisms of POD.

## RESULTS

### Surgery/Anesthesia impaired the behavior of aged mice at 6, 9, and 24 hours postoperatively

**Figure 1:**
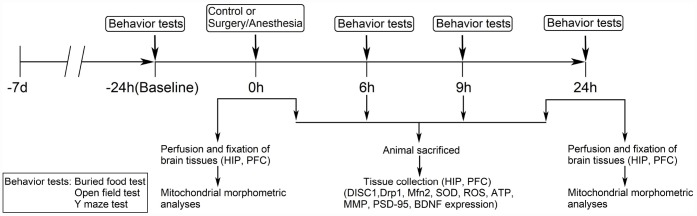
**Diagram of the experimental design.** The mice underwent behavioral tests at 24 hours before (baseline) and at 6, 9, and 24 hours after the Surgery/Anesthesia. Within each group, separate cohorts were subjected to assessments at each time point (n = 9 per cohort). Mice were sacrificed immediately after the Surgery/Anesthesia and at 6, 9, and 24 hours postoperatively. The hippocampal and prefrontal cortex tissues were harvested for analysis of DISC1, Drp1, Mfn2, SOD, ROS, ATP, MMP, BDNF, and PSD-95 levels (n = 6 per cohort). Mice were anesthetized and transcardially perfused with ice-cold phosphate-buffered saline (PBS) followed by paraformaldehyde and glutaraldehyde; then, hippocampal and prefrontal cortex tissues were collected and stored in the same fixative for electron microscopy analysis immediately after the Surgery/Anesthesia and at 24 hours postoperatively (n = 3 per cohort). DISC1, disrupted in schizophrenia 1. Drp1, dynamin-related protein 1. Mfn2, mitofusin 2. SOD, superoxide dismutase. ROS, reactive oxygen species. ATP, adenosine triphosphate. MMP, mitochondrial membrane potential. PSD-95, postsynaptic density protein 95. BDNF, brain-derived neurotrophic factor.

We set out to assess the effects of Surgery/Anesthesia on aged mice behavior in the buried food test, open field test, and Y maze test at 24 hours before the procedure and then 6, 9, or 24 hours postoperatively (Figure 1).

**Figure 2:**
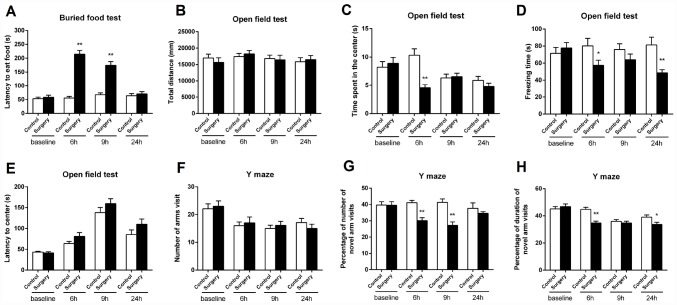
**Surgery/Anesthesia impaired the behavior of aged mice at 6, 9, and 24 hours postoperatively.** (**A**) Surgery/Anesthesia increased the latency of mice to eat the food as compared to the control condition in the buried food test at 6 and 9 hours postoperatively. Surgery/Anesthesia did not significantly alter the latency of mice to eat food as compared to the control condition at 24 hours postoperatively. (**B**) Surgery/Anesthesia did not significantly change the total distance travelled by mice in the open field test as compared to the control condition at 6, 9, and 24 hours postoperatively. (**C**) Surgery/Anesthesia significantly decreased the time spent in the center of the open field as compared to the control condition at 6 but neither 9 nor 24 hours postoperatively. (**D**) Surgery/Anesthesia significantly decreased the freezing time in the open field test as compared to the control condition at 6 and 24 but not 9 hours postoperatively. (**E**) Surgery/Anesthesia did not significantly change the time to reach the center (latency to the center) in the open field test as compared to the control condition at 6, 9, and 24 hours postoperatively. (**F**) Surgery/Anesthesia did not significantly change the number of arm visits in the Y maze test as compared to the control condition at 6, 9, and 24 hours postoperatively. (**G**) Surgery/Anesthesia significantly decreased the number of entries in the novel arm in the Y maze test as compared to the control condition at 6 and 9 but not 24 hours postoperatively. (**H**) Surgery/Anesthesia significantly decreased the duration in the novel arm in the Y maze test as compared to the control condition at 6 and 24 but not 9 hours postoperatively. The data are plotted as the mean ± standard error of the mean for each group (n = 9). ^*^p < 0.05 and ^**^p < 0.01, compared to control.

We first assessed whether Surgery/Anesthesia could impair the natural behaviors of aged mice using the buried food test. As shown in Figure 2A, Surgery/Anesthesia increased the latency of mice to eat the food as compared to the control condition at 6 and 9 hours (*p* < 0.01) but not 24 hours postoperatively. These data suggest that Surgery/Anesthesia may impair the mice’s abilities to find and eat the food, and this impairment was time-dependent.

Then, we assessed the effects of Surgery/Anesthesia on the open field behavior in the aged mice. Surgery/Anesthesia did not significantly change the total distance travelled by mice as compared to the control condition at 6, 9, and 24 hours postoperatively (Figure 2B). These data suggest that the Surgery/Anesthesia did not impair the motor function of the mice. As compared to the control condition, Surgery/Anesthesia significantly decreased the time spent in the center at 6 (*p* < 0.01) but neither 9 nor 24 hours postoperatively in mice (Figure 2C). Surgery/ Anesthesia also significantly decreased the freezing time as compared to the control condition at 6 (*p* < 0.05) and 24 (*p* < 0.01) but not 9 hours postoperatively in mice (Figure 2D). However, Surgery/Anesthesia did not significantly change the time to reach the center (latency to the center) as compared to the control condition at all the time points (Figure 2E). In conclusion, these data suggest that Surgery/Anesthesia could affect several open field behaviors (e.g., time spent in the center and freezing time), but not others (e.g., total distance and latency to the center), in mice in a time-dependent manner.

Finally, we assessed whether Surgery/Anesthesia could impair learned behavior in aged mice by employing the Y maze test. As can be seen in Figure 2F, Surgery/Anesthesia did not significantly change the number of arm visits as compared to the control condition at all the postoperative time points. These data showed again that the Surgery/Anesthesia did not impair the motor functions of the mice. Surgery/Anesthesia significantly decreased the number of entries in the novel arm as compared to the control condition at 6 (*p* < 0.01) and 9 (*p* < 0.01) but not 24 hours postoperatively in mice. Besides, Surgery/Anesthesia significantly decreased the duration in the novel arm as compared to the control condition at 6 (*p* < 0.01) and 24 (*p* < 0.05) but not 9 hours postoperatively in mice. Collectively, these data suggest that Surgery/Anesthesia could disturb some Y maze behaviors (e.g., entries in the novel arm and duration in the novel arm), but not others (e.g., number of arm visits), in mice, and such disturbances were time-dependent.

Taken together, Surgery/Anesthesia impaired the natural (buried food test and open field test) and learned (Y maze test) behaviors of aged mice in an acute and fluctuating manner (Figure 2).

### Surgery/Anesthesia decreased DISC1 expression in the hippocampus and prefrontal cortex of aged mice at 0, 6, 9, and 24 hours postoperatively

**Figure 3:**
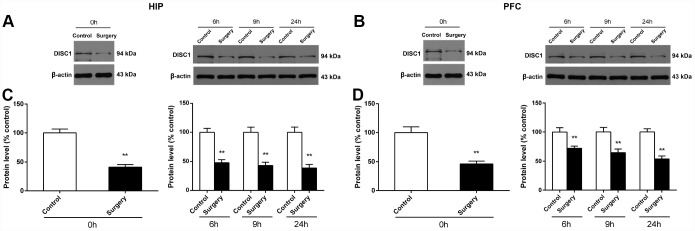
**Surgery/Anesthesia decreased the DISC1 expression in the hippocampus and prefrontal cortex of aged mice at postoperative 0, 6, 9, and 24 hours.** The expression of DISC1 was estimated using western blotting analysis of fresh homogenates from hippocampal and prefrontal cortex tissues of mice at 0, 6, 9, and 24 hours after Surgery/Anesthesia or control treatment. (**A** and **C**) The level of DISC1 in the hippocampus of mice in the Surgery/Anesthesia group decreased significantly compared to that in control mice at postoperative 0, 6, 9, and 24 hours. (**B** and **D**) The level of DISC1 in the prefrontal cortex tissue of mice in the Surgery/Anesthesia group decreased significantly compared to that in control mice at postoperative 0, 6, 9, and 24 hours. The data are plotted as the mean ± standard error of the mean for each group (n = 6). ^*^p < 0.05 and ^**^p < 0.01, compared to control.

It has been reported that the inhibition of DISC1 may lead to the down-regulation of Drp1 and thus regulate mitochondrial dynamics [18]. We measured the level of DISC1 in the hippocampus and prefrontal cortex of mice at 0, 6, 9, and 24 hours after Surgery/Anesthesia using western blot. As shown in Figure 3, DISC1 expression in the Surgery/Anesthesia mice decreased significantly compared to that in control mice both in the hippocampus (*p* < 0.01) (Figure 3C) and the prefrontal cortex (*p* < 0.01) (Figure 3D) at postoperative 0, 6, 9, and 24 hours.

### Surgery/Anesthesia changed the expression of Drp1 and Mfn2 in the cytosolic fraction and the mitochondrial fraction and caused ultrastructural changes in the hippocampus and prefrontal cortex of aged mice at 0, 6, 9, and 24 hours postoperatively

We assessed whether Surgery/Anesthesia modulates the expression of two key proteins, Drp1 and Mfn2, responsible for maintaining mitochondrial dynamics. These two mitochondrial dynamics related proteins are localized in the cytoplasm or sequestered in the mitochondrial membrane and may be involved in active remodeling of the outer and inner mitochondrial membranes. Accordingly, the expression of Drp1 and Mfn2 in both the cytosolic and mitochondrial compartments were measured.

**Figure 4:**
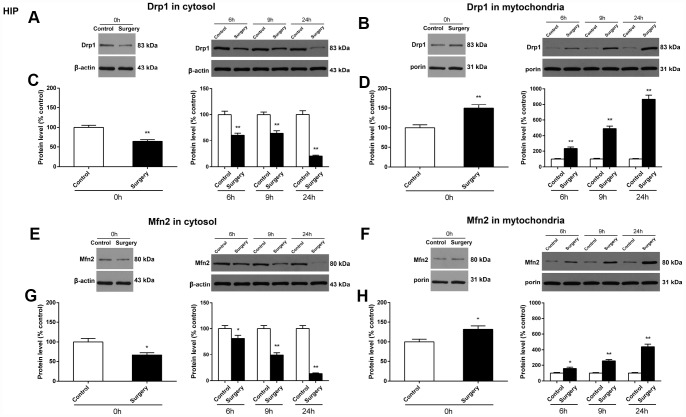
**Surgery/Anesthesia changed the expression of Drp1 and Mfn2 in the cytosolic fraction and in the mitochondrial fraction of the hippocampus in aged mice at 0, 6, 9, and 24 hours postoperatively.** The expression of Drp1 and Mfn2 was estimated using western blotting analysis of fresh cytosolic and mitochondrial fractions of the hippocampus obtained from mice at 0, 6, 9, and 24 hours after Surgery/Anesthesia or control treatment. As compared to control condition in mice, Surgery/Anesthesia decreased Drp1 expression in the cytosolic fraction (**A** and **C**) and increased Drp1 expression in the mitochondrial fraction (**B** and **D**) of homogenates of hippocampal tissue. When compared to the control condition in mice, Surgery/Anesthesia significantly decreased the expression of Mfn2 in the cytosolic fraction (**E** and **G**) and increased Mfn2 level in the mitochondrial fraction (**F** and **H**) of homogenates of hippocampus tissue at 0, 6, 9, and 24 hours postoperatively. The data are plotted as the mean ± standard error of the mean for each group (n = 6). ^*^p < 0.05 and ^**^p < 0.01, compared to control.

In the hippocampus of mice, Surgery/Anesthesia decreased Drp1 expression in the cytosolic fraction (*p* < 0.01) (Figure 4C) and increased Drp1 expression in the mitochondrial fraction (*p* < 0.01) (Figure 4D) as compared to the control condition at 0, 6, 9, and 24 hours postoperatively. Likewise, Surgery/Anesthesia significantly decreased the expression of Mfn2 in the cytosolic fraction (*p* < 0.05 for 0 hours, *p* < 0.05 for 6 hours, *p* < 0.01 for 9 hours, and *p* < 0.01 for 24 hours) (Figure 4G) and increased Mfn2 expression in the mitochondrial fraction (*p* < 0.05 for 0 hours, *p* < 0.05 for 6 hours, *p* < 0.01 for 9 hours, and *p* < 0.01 for 24 hours) (Figure 4H) when compared to control condition at all the postoperative time points.

**Figure 5:**
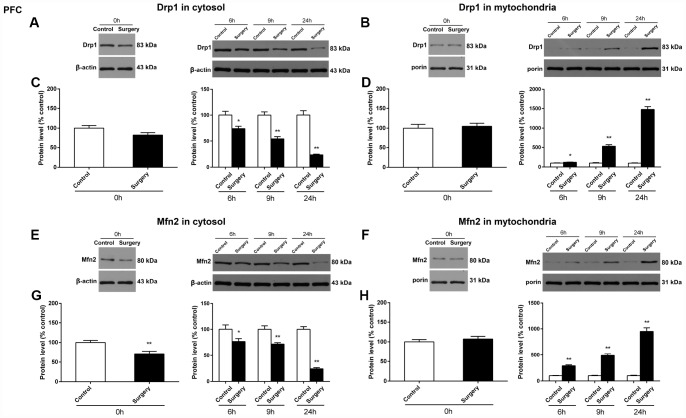
**Surgery/Anesthesia changed the expression of Drp1 and Mfn2 in the cytosolic fraction and in the mitochondrial fraction of the prefrontal cortex tissues in aged mice at postoperative 0, 6, 9, and 24 hours.** Immediately after Surgery/Anesthesia, there was no significant change in the expression of Drp1 both in the cytosolic fraction (**A** and **C**) and the mitochondrial fraction (B and D) as compared to that in the control condition. At 6, 9, and 24 hours postoperatively, Surgery/Anesthesia significantly decreased the expression of Drp1 in the cytosolic fraction (**A** and **C**) and increased Drp1 protein level in the mitochondrial fraction (**B** and **D**) from the prefrontal cortex tissues in mice when compared to the control condition. Additionally, Surgery/Anesthesia decreased the expression of Mfn2 in the cytosolic fraction (**E** and **G**) at 0, 6, 9, and 24 hours postoperatively. When compared to the control condition, Surgery/Anesthesia did not significantly change the expression of Mfn2 in the mitochondrial fraction (**F** and **H**) at 0 hour postoperatively. As compared to the control condition, Surgery/Anesthesia significantly increased Mfn2 expression in the mitochondrial fraction (**F** and **H**) from the prefrontal cortex tissue in mice at 6, 9, and 24 hours postoperatively. The data are plotted as the mean ± standard error of the mean for each group (n = 6). ^*^p < 0.05 and ^**^p < 0.01, compared to control.

In the prefrontal cortex of mice, as compared to the control condition, Surgery/Anesthesia did not significantly change the expression of Drp1 both in the cytosolic fraction (Figure 5C) and the mitochondrial fraction (Figure 5D) immediately after Surgery/Anesthesia. However, Surgery/Anesthesia significantly decreased Drp1 expression in the cytosolic fraction (Figure 5C) and increased Drp1 expression in the mitochondrial fraction (Figure 5D) when compared to control condition at 6 (*p* < 0.05), 9 (*p* < 0.01), and 24 (*p* < 0.01) hours postoperatively. Additionally, as compared to the control condition, Surgery/Anesthesia significantly decreased the Mfn2 expression in the cytosolic fraction in mice at 0 (*p* < 0.01), 6 (*p* < 0.05), 9 (*p* < 0.01), and 24 (*p* < 0.01) hours postoperatively (Figure 5G). Furthermore, as compared to the control condition, Surgery/Anesthesia significantly increased Mfn2 expression in the mitochondrial fraction in mice at 6, 9, and 24 hours (*p* < 0.01) but not immediately after Surgery/Anesthesia (Figure 5H).

**Figure 6:**
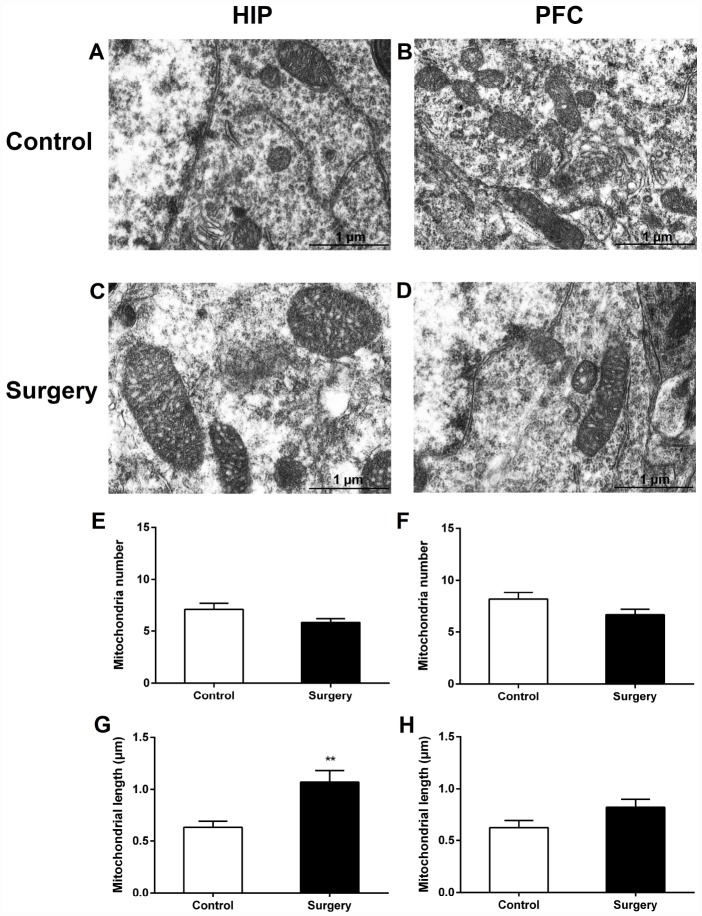
**Surgery/Anesthesia caused acute ultrastructural changes in the mitochondria of hippocampal but not prefrontal cortex neurons in aged mice immediately after Surgery/Anesthesia.** Mitochondria in the cytoplasm of hippocampal (**A**) and prefrontal cortex (**B**) neurons from the control mice resemble long tubules with intact outer and inner membranes and numerous cristae tightly packed in healthy looking matrix. Compared with those in the control group, mitochondria in the cytoplasm of hippocampal neurons (**C**) from mice in the Surgery/Anesthesia group became swollen, while the ultrastructure of mitochondria in the prefrontal cortex neurons (**D**) was normal immediately after Surgery/Anesthesia. The number and length of mitochondria were measured in the hippocampus (**E**, **G**) and prefrontal cortex (**F**, **H**) in 6 different fields of view per animal. (**G**) Surgery/Anesthesia increased mitochondrial length in the hippocampus compared to the control condition at 0 hour postoperatively. Scale bar: 1 μm. The data are plotted as the mean ± standard error of the mean for each group (n = 3). ^**^p < 0.01, compared to control.

The ultrastructural analysis of mitochondrial morphology in the hippocampal and prefrontal cortex neurons revealed that Surgery/Anesthesia caused acute ultrastructural changes in the mitochondria of hippocampal neurons in aged mice immediately after Surgery/Anesthesia. Compared to those in the control condition (Figure 6A), immediately after Surgery/Anesthesia the mitochondria in the cytoplasm of hippocampal neurons from mice in the Surgery/Anesthesia group (Figure 6C) were swollen, and the mitochondrial length increased significantly (*p* < 0.01) (Figure 6G). However, the ultrastructure of mitochondria in the prefrontal cortex neurons of surgical mice immediately after Surgery/Anesthesia (Figure 6D) had no significant changes as compared with that of control mice (Figure 6B).

**Figure 7:**
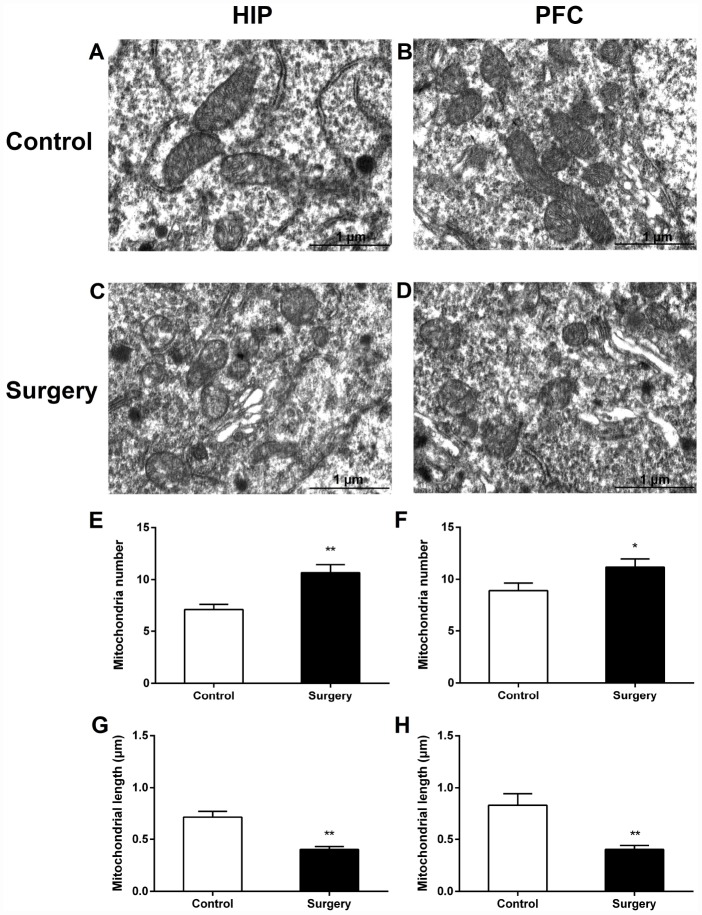
**Surgery/Anesthesia caused ultrastructural changes in the mitochondria of hippocampal and prefrontal cortex neurons in aged mice at 24 hours postoperatively.** Mitochondria in the cytoplasm of hippocampal (**A**) and prefrontal cortex (**B**) neurons from the control mice resemble long tubules with intact outer and inner membranes and numerous cristae tightly packed in healthy looking matrix. The number of mitochondria in the cytoplasm of hippocampal (**C** and **E**) and prefrontal cortex (**D** and **F**) neurons from mice in the Surgery/Anesthesia group were increased significantly. Compared to the control condition, Surgery/ Anesthesia decreased the mitochondrial length in the hippocampus (**G**) and prefrontal cortex (**H**) at 24 hours postoperatively. The mitochondria in the Surgery/Anesthesia group were small, round, and displayed globular morphology. Although the outer and inner membranes appeared somewhat intact, the cristae seemed distorted and difficult to discern. Scale bar: 1 μm. The data are plotted as the mean ± standard error of the mean for each group (n = 3). ^*^p < 0.05 and ^**^p < 0.01, compared to control.

At 24 hours postoperatively, Surgery/Anesthesia caused ultrastructural changes in the mitochondria of both hippocampal and prefrontal cortex neurons. Compared to that in the control mice, the number of mitochondria in the cytoplasm of hippocampal (Figure 7C, 7E) and prefrontal cortex (Figure 7D, 7F) neurons in mice in the Surgery/Anesthesia group significantly increased, and the mitochondrial length significantly decreased (Figure 7G, 7H) (*p* < 0.01). The mitochondria in the Surgery/ Anesthesia group were small, round, and displayed globular morphology. Although the outer and inner membranes appeared somewhat intact, the cristae seemed distorted and difficult to discern, suggesting ultrastructural damage to mitochondria undergoing excessive fission.

### Surgery/Anesthesia increased the level of ROS and decreased the level of SOD in the hippocampus and prefrontal cortex of aged mice at 0, 6, 9, and 24 hours postoperatively

**Figure 8:**
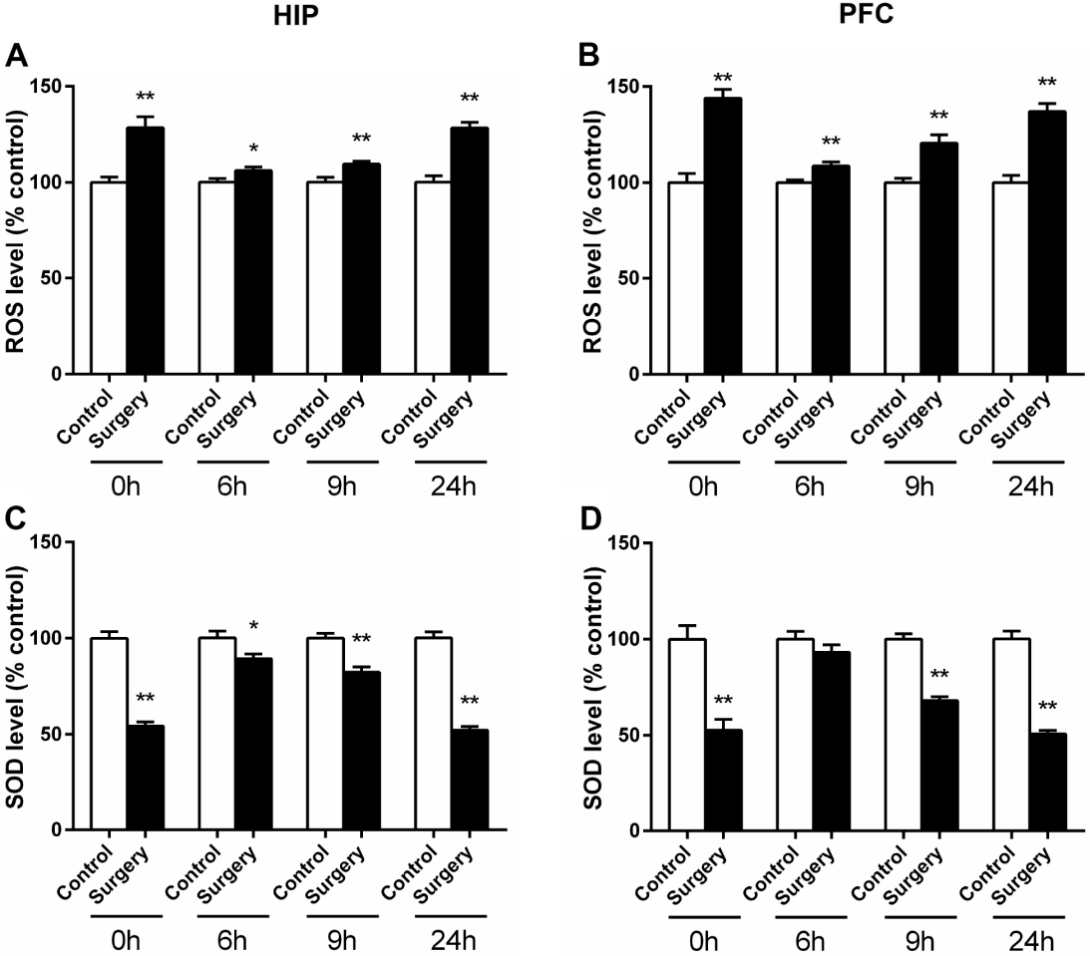
**Surgery/Anesthesia altered the activity level of SOD and ROS in the hippocampus and prefrontal cortex of aged mice at 0, 6, 9, and 24 hours postoperatively.** SOD and ROS levels were measured in fresh homogenates from hippocampal and prefrontal cortex tissues of mice at 0, 6, 9, and 24 hours after Surgery/Anesthesia or control treatment. The level of ROS in the hippocampus (**A**) and prefrontal cortex (**B**) of mice in the Surgery/Anesthesia group increased significantly compared to that in the control mice at all the postoperative timepoints. Conversely, the SOD level in the hippocampus (**C**) of mice in the Surgery/Anesthesia group decreased significantly compared to that in control mice at 0, 6, 9, and 24 hours postoperatively. (**D**) The level of SOD in the prefrontal cortex tissues of mice in the Surgery/Anesthesia group decreased significantly as compared to that in control mice at 0, 9, and 24 hours but not 6 hours postoperatively. The data are plotted as the mean ± standard error of the mean for each group (n = 6). ^*^p < 0.05 and ^**^p < 0.01, compared to control.

Excessive production of ROS induced oxidative stress response was reported to play an important role in the pathogenesis of POD [10, 13]. Mitochondria are well known as the main source of ROS through one-electron carriers in the respiratory chain [19] and are the major targets of free radical attack [20]. In addition, precipitous mitochondrial fission or fragmentation may accompany ROS production and oxidative damage [17]. Therefore, we set out to investigate whether Surgery/ Anesthesia causes undue accumulation of ROS. As can be seen in Figure 8A, the level of ROS in the hippocampus of mice in Surgery/Anesthesia group increased significantly at 0 (*p* < 0.01), 6 (*p* < 0.05), 9 (*p* < 0.01), and 24 (*p* < 0.01) hours postoperatively when compared to that in the control mice. As shown in Figure 8B, as compared to the control condition, Surgery/Anesthesia also significantly increased the level of ROS in the prefrontal cortex tissues in mice at all the postoperative time points (*p* < 0.01). These data suggested that Surgery/Anesthesia significantly promotes ROS generation in the brain of aged mice.

SOD is widely known as a marker of anti-oxidative stress, which can scavenge excessive ROS and thus play a crucial role in maintaining cellular redox homeostasis [21, 22]. We measured SOD level in brain homogenate from the hippocampus and prefrontal cortex of aged mice at 0, 6, 9, and 24 hours postoperatively. As shown in Figure 8C, the level of SOD in the hippocampus of mice in the Surgery/Anesthesia group decreased significantly at 0 (*p* < 0.01), 6 (*p* < 0.05), 9 (*p* < 0.01), and 24 (*p* < 0.01) hours postoperatively compared to that in the control condition. As can be seen in Figure 8D, the level of SOD in the prefrontal cortex tissues of mice in the Surgery/Anesthesia group decreased significantly as compared to that in control mice at 0, 9, and 24 hours (*p* < 0.01) but not 6 hours postoperatively. These results indicated that Surgery/ Anesthesia impaired SOD activity in the brain of aged mice. Taken together, oxidative stress response was elevated and anti-oxidative stress ability was impaired postoperatively.

### Surgery/Anesthesia decreased the levels of MMP and ATP in the hippocampus and prefrontal cortex of aged mice at 0, 6, 9, and 24 hours postoperatively

**Figure 9:**
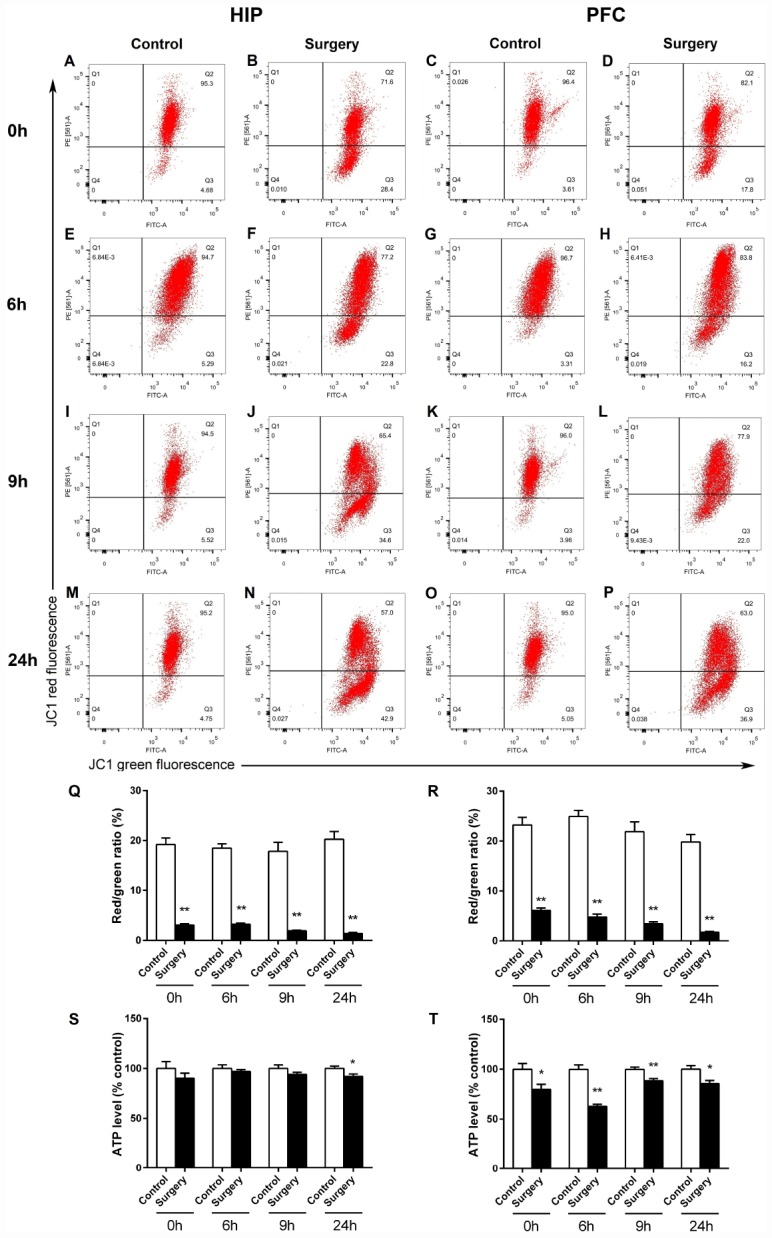
**Surgery/Anesthesia altered the levels of MMP and ATP in the hippocampus and prefrontal cortex of aged mice at 0, 6, 9, and 24 hours postoperatively.** Changes in MMP were measured using flow cytometry and JC-1. Representative graphs of flow cytometric analysis of the altered MMP level in the hippocampus (**A**, **B**, **E**, **F**, **I**, **J**, **M**, **N**) and prefrontal cortex (**C**, **D**, **G**, **H**, **K**, **L**, **O**, **P**) of mice after incubation with JC-1. Statistical bar graphs show the changes of MMP detected using flow cytometry. The changes of MMP in the hippocampal (**Q**) and prefrontal cortex tissues (**R**) were defined as the ratio of red/green fluorescence intensity. Surgery/Anesthesia reduced the MMP level in the hippocampus and prefrontal cortex as compared to the control condition in mice immediately after Surgery/ Anesthesia. (**S**) Surgery/Anesthesia decreased the ATP level in the hippocampus at 24 hours but not at 0, 6, or 9 hours postoperatively. (**T**) In the prefrontal cortex, Surgery/Anesthesia significantly decreased the level of ATP as compared to control condition in mice at all the postoperative timepoints. The data are plotted as the mean ± standard error of the mean for each group (n = 6). ^*^p < 0.05 and ^**^p < 0.01, compared to control.

MMP and ATP are recognized as indicators of mitochondrial activity, and the stability of MMP plays a crucial function in ATP generation [23–25]. The cyanine dye 5,5,6,6’-tetrachloro-1,1’,3,3’ tetraethylbenzimidazolylcarbocyanine iodide (JC-1) and flow cytometry were utilized to assess the changes in MMP. As shown in Figure 9, Surgery/Anesthesia reduced MMP level in the hippocampus and prefrontal cortex as compared to the control condition in mice at 0, 6, 9, and 24 hours postoperatively (*p* < 0.01) (Figure 9A–9R).

Mitochondria are reported to play important roles in ATP production, and ATP level is a sensitive readout of mitochondrial function [17, 25]. We measured ATP levels in fresh brain homogenates from the hippocampus and prefrontal cortex of aged mice at 0, 6, 9, and 24 hours postoperatively. As shown in Figure 9S, Surgery/Anesthesia decreased the ATP level in the hippocampus at 24 (*p* < 0.05) but not at 0, 6, or 9 hours postoperatively. In the prefrontal cortex tissue, Surgery/Anesthesia decreased the ATP level at 0 (*p* < 0.05), 6 (*p* < 0.01), 9 (*p* < 0.01), and 24 (*p* < 0.05) hours after Surgery/Anesthesia as compared to the control condition in mice (Figure 9T). These findings indicate that Surgery/Anesthesia impaired the mitochondrial functions of MMP maintenance and ATP synthesis in the brain of aged mice.

### Surgery/Anesthesia decreased the expression of PSD-95 and BDNF in the hippocampus and prefrontal cortex of aged mice at 0, 6, 9, and 24 hours postoperatively

**Figure 10:**
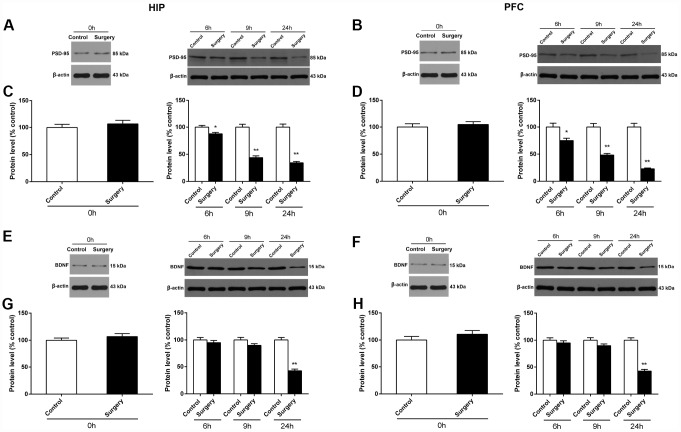
**Surgery/Anesthesia decreased PSD-95 and BDNF expression in the hippocampus and prefrontal cortex of aged mice at postoperative 0, 6, 9, and 24 hours.** The expression of PSD-95 and BDNF was estimated using western blotting analysis of fresh homogenates of hippocampal and prefrontal cortex tissues obtained at 0, 6, 9, and 24 hours after Surgery/Anesthesia or control treatment. Immediately after Surgery/Anesthesia, there were no significant differences in the PSD-95 expression in the hippocampus (**A** and **C**) and prefrontal cortex (**B** and **D**) between the mice in the Surgery/Anesthesia group and control group. However, the level of PSD-95 in the hippocampus (**A** and **C**) and prefrontal cortex (**B** and **D**) of mice in the Surgery/Anesthesia group decreased significantly compared to that in control mice at postoperative 6, 9, and 24 hours. Additionally, Surgery/Anesthesia decreased the BDNF expression in the hippocampus (**E** and **G**) and the prefrontal cortex tissues (**F** and **H**) at 24 but not 0, 6, or 9 hours postoperatively when compared to the control condition in mice. The data are plotted as the mean ± standard error of the mean for each group (n = 6). ^*^p < 0.05 and ^**^p < 0.01, compared to control.

PSD-95, a major postsynaptic scaffold protein [26, 27], is reported to play a key role in modulating synaptic function and plasticity [27–29]. BDNF is a growth factor that serves essential functions in synaptic plasticity and neuronal differentiation [30, 31]. We measured the levels of PSD-95 and BDNF in the hippocampus and prefrontal cortex at 0, 6, 9, and 24 hours after Surgery/Anesthesia using western blot. The level of PSD-95 in the hippocampus (Figure 10C) and prefrontal cortex (Figure 10D) of mice in the Surgery/Anesthesia group decreased significantly, compared to that of control mice at postoperative 6 (*p* < 0.05), 9 (*p* < 0.01), and 24 (*p* < 0.01) hours, but not immediately after Surgery/Anesthesia. In addition, Surgery/Anesthesia decreased the BDNF expression in the hippocampus (Figure 10G) and the prefrontal cortex tissues (Figure 10H) at 24 *(p* < 0.01) but not 0, 6, or 9 hours postoperatively when compared to the control condition in mice. These findings provide direct evidence that Surgery/Anesthesia decreased the expression of PSD-95 and BDNF in the hippocampus and prefrontal cortex of aged mice.

## DISCUSSION

The goal of this study was to investigate the effects of Surgery/Anesthesia on mitochondrial fission/fusion dynamics in the brain of aged mice with POD.

We used Confusion Assessment Method (CAM)-in mice battery (consists of buried food test, open field test, and Y maze test), which was described in our previous study [32], to assess behavioral changes after Surgery/Anesthesia in aged mice and found that abdominal surgery under isoflurane anesthesia induced an acute and fluctuating delirium-like behavior in aged mice. In addition, we demonstrated that Surgery/Anesthesia could disturb mitochondrial fission/fusion dynamics in the brain of aged mice with POD. Moreover, we revealed the impact of Surgery/Anesthesia -derived abnormal mitochondrial fission/fusion dynamics on mitochondrial function (e.g. disruption of the balance between the generation and depletion of ROS, with excessive accumulation of ROS and impaired SOD activity, and defects in energy metabolism, with reduced MMP levels and decreased ATP levels) as well as the decreased expression of neuronal/synaptic plasticity-related proteins (e.g. decreased BDNF and PSD-95 levels). To our knowledge, this is the first evaluation of the influence of Surgery/Anesthesia on brain mitochondrial fission/fusion, mitochondrial function, synapse-associated proteins expression, as well as delirium-like behavior in aged mice.

Drp1 and Mfn2 are two key GTPase proteins responsible for maintaining mitochondrial fission and fusion dynamics [15, 33]. Drp1 is mainly localized in the cytosol and is the key regulator in the mitochondrial fission process. During fission, cytosolic Drp1 is recruited to the mitochondrial outer membrane, followed by its oligomerization into a ring-like structure to sever the mitochondrial membrane by self-assembly and GTP hydrolysis [34, 35]. Mfn2 regulates mitochondrial outer membrane fusion. It has been proposed that interactions of the coiled-coil domains of Mfn1 and Mfn2 could form either homo-oligomeric or hetero-oligomeric complexes to tether membranes together [36]. In the current study, we found that Surgery/Anesthesia induced an acute decrease of Drp1 expression in the cytosolic fraction as well as increased Drp1 expression in the mitochondrial fraction of tissue homogenate from the hippocampus and prefrontal cortex, suggesting Drp1 translocation into mitochondria and excessive mitochondrial fission. Furthermore, the ultrastructural analysis of mitochondrial morphology in hippocampal and prefrontal cortex neurons also showed the excessive fission of mitochondria. As we know, mitochondrial fusion may protect their function by mixing the mitochondrial contents, which enables mitochondrial DNA repair, protein complementation, and proper distribution of metabolites [37]. In our study, expression of the mitochondrial fusion related protein Mfn2 increased in the mitochondrial fraction; however, mitochondrial morphology showed excessive fission. This is not surprising, as it could be possible that mitochondrial fission and fusion play important roles in mitochondrial quality control for preserving mitochondria [38], and the protein level of Mfn2 may be upregulated in the mitochondrial fraction as a compensatory mechanism to prevent harmful defects [39], such as the upregulation of ROS and mitochondrial fragmentation [40, 41]. DISC1 was reported to affect mitochondrial dynamics by upregulating Drp1 expression in glioblastoma cells [18]. In the present study, we found that Surgery/Anesthesia induced an acute decrease in DISC1 expression and decreased Drp1 expression in the cytosolic fraction. It is possible that Surgery/Anesthesia impaired mitochondrial function by inhibiting DISC1 expression, leading to increased Drp1 levels in mitochondria (Drp1 translocation to mitochondria), and then promoting excessive mitochondrial fission and mitochondrial fragmentation.

The imbalance of mitochondrial fission/fusion dynamics can lead to mitochondrial dysfunction [42, 43], including excessively activated oxidative stress and energy deficits. Surgery/Anesthesia caused disruptions in the balance between the generation and depletion of ROS, including the upregulation of ROS levels and the downregulation of SOD. It has been reported that impaired mitochondria are not only powerful sources of ROS but also an important target for them [44, 45]. On one hand, excessive mitochondrial fragmentation causes ROS overproduction [46]; on the other hand, oxidative stress impairs mitochondrial function by inducing structural changes [47]. SOD serves as an efficient scavenger of ROS [48], and the aging brain might be more vulnerable to oxidative stress due to the normal antioxidant defense mechanisms declining with age [49, 50]. Increasing evidence demonstrates that abnormalities in oxidative stress play an important role in the neuropathogenesis of POD [10, 13]. Our data also showed that Surgery/Anesthesia induced a significant increase in ROS level and decrease in SOD level, indicating that excessive activation of oxidative stress and free radical scavenging disorders are likely to be involved in the pathogenesis of POD. As we know, the brain is a high energy consuming organ with high metabolic activity [51], and neurons rely almost exclusively on the mitochondrial oxidative phosphorylation (OXPHOS) system to fulfill their energy needs [52]. Mitochondria are important in energy metabolism due to their function in ATP generation, which is reliant on the stability of MMP [24]. Loss of MMP could open the mitochondrial permeability transition pore (mPTP) and then lead to osmotic swelling of the mitochondrial matrix and defective OXPHOS, thus impairing ATP synthesis [23]. Xu et al [53] showed a reduction in ATP level and MMP in mice with anesthesia-induced cognitive dysfunction, suggesting the role of energy deficits in cognitive decline. Our previous studies also demonstrated that Surgery/Anesthesia may induce energy deficits in brain tissues, whereas employing the mPTP opening inhibitor cyclosporine A could attenuate the Surgery/Anesthesia-induced reduction in ATP levels and mitigate certain delirium-like behavior in mice [32]. In the present study, ATP level and MMP were decreased in the hippocampus and prefrontal cortex of aged mice, implying that mitochondrial dysfunction-related brain energy deficits also contribute to the Surgery/Anesthesia-induced POD.

Mitochondrial functioning may impact brain functions, such as cognition, by influencing synaptic transmission [54]. Neuronal/synaptic plasticity related proteins such as PSD-95 and BDNF are involved in synaptic transmission and play an important role in learning and memory [55, 56]. The synaptic marker PSD-95 plays an important role in modulating synaptic function and plasticity [27, 28, 57]. PSD-95 expression in the hippocampus was found to be decreased in mice that underwent surgery and anesthesia, while increased expression of PSD-95 may contribute to the improvement of the surgery-induced cognitive impairments [53, 58]. BDNF is a growth factor that serves essential functions in synaptic plasticity and neuronal differentiation [30, 31, 59]. The reduction of BDNF levels may impair neurogenesis and neuronal plasticity as well as learning and memory [59, 60], which could account, at least in part, for the pathogenesis of surgery-induced cognitive dysfunction [61]. On the contrary, an increased BDNF level is associated with the improvement in emotional and spatial memories [62]. Our data suggested that the decreased expression of the neuronal/synaptic plasticity related proteins PSD-95 and BDNF might be related to mitochondrial dysfunction and delirium-like behavior, which are induced by Surgery/Anesthesia.

Collectively, all of these results suggested that Surgery/Anesthesia may disturb mitochondrial fission/fusion dynamics, lead to mitochondrial dysfunction, which includes excessive oxidative stress and energy deficits, and then downregulate the expression of neuronal/synaptic plasticity related proteins, contributing to the pathogenesis of POD.

Our previous studies have suggested that mitochondrial dysfunction could contribute to POD [32]. In that study, we used 4-month-old adult mice and found that Surgery/Anesthesia only altered the natural and learned behaviors at 6 or 9 hours but not 24 hours postoperatively, suggesting acute onset of the behavioral changes. In addition, Zhang et al [13] used 2-month-old adult mice and also found that significant effects of surgery under 1.4% isoflurane anesthesia on behavior, such as latency to eat food and time spent in the center, occurred at 6 or 9 hours after operation but disappeared after 24 hours. However, in the present study, the impaired behaviors of mice were not fully recovered at 24 hours after Surgery/Anesthesia. The inconsistency of these results may be related to the different age of the mice, as 18-month-old aged mice were used in the current study. The discrepancy also supports the notion that, in mice, aging is critically involved in the development of POD. Aged mice are more vulnerable to the development of cognitive impairment following surgery [63]. The recovery of cognitive function of aged mice after Surgery/Anesthesia could be slower due to the lack of physiologic reserves [10, 11], declined neuroplasticity and angiogenesis [64], and impaired mitochondrial function [49, 50].

The current study has several limitations. First, considering that the elderly are more susceptible to POD and that the current study was a pilot study to assess the role of mitochondrial dynamics in the neuropathogenesis of POD, we only employed 18-month-old aged mice but did not investigate the influence of Surgery/Anesthesia on the mitochondrial fission/fusion dynamics in the brain of adult mice. Future research would include the comparison of the effects of Surgery/Anesthesia on mitochondrial fission/fusion dynamics and behavioral changes in mice of different ages. Second, we only assessed the effects of Surgery/Anesthesia on mitochondrial fission/fusion dynamics in the hippocampus and prefrontal cortex of mice. The hippocampus is involved in emotions, learning, and memory formation [65], and the prefrontal cortex encodes task relevant information in working memory [66, 67]. Moreover, the hippocampus and prefrontal cortex interact during memory formation, consolidation, and expression [68]. However, Surgery/Anesthesia could have different effects on mitochondrial fission/fusion dynamics in different regions of the brain, such as the amygdala, thalamus, and cingulate cortex. Therefore, future investigations should look into the potential effects of Surgery/Anesthesia on mitochondrial dynamics in other regions of the brain. Third, there are three subtypes of delirium in clinical setting—hyperactive, hypoactive, and mixed. In the present study, the CAM-in mice battery was used only to test the delirium-like behaviors, such as inattention, disorganized thinking, and decreased level of consciousness, but not to identify the subtype of delirium. To better understand the mechanism of POD in future studies, POD animal models with subtype diagnosis are needed. Fourth, in the current study, we considered anesthesia and surgery as a whole to mimic the clinical setting, considering that patients receiving surgery have to experience anesthetic treatment, and did not set up a group subjected only to isoflurane anesthesia. However, anesthesia itself has been reported to disturb mitochondrial fission/fusion dynamics, induce mitochondrial dysfunction, and impair learning and memory in rodents [40, 69, 70]. Thus, we cannot rule out the effect of anesthesia on mitochondrial dynamics and delirium-like behavior in mice. Further studies will examine the effects of isoflurane anesthesia or other anesthetics on mitochondrial fission/fusion dynamics and delirium-like behavioral changes in the brain of aged mice. Finally, we did not apply the live imaging of neuron/ synapse morphology, which may need to be considered in future investigations for the exploration of the underlying mechanisms of mitochondrial dynamics.

In conclusion, we found that abdominal surgery under isoflurane anesthesia (Surgery/Anesthesia) may contribute to POD via mitochondrial dysfunction-related oxidative stress and energy deficits induced by impaired mitochondrial fission/fusion dynamics in the hippocampus and prefrontal cortex of aged mice, providing new insights for investigations into the pathogenesis of POD.

## MATERIALS AND METHODS

### Mice

All procedures were approved by the Animal Ethics Committee of Zhongnan Hospital of Wuhan University, Wuhan, China, and all experiments were performed in accordance with the National Institutes of Health Guidelines for the Care and Use of Laboratory Animals. Efforts were made to minimize the number of animals used. C57BL/6 female mice (Changsha Tianqin Biotechnology Co., Ltd.; Changsha, China) (18-month-old, weighing 30–40 g) were used in this study. All animals were housed five per cage with free access to food and water. The temperature, humidity, and day-night cycle were maintained according to the standards established by the experimental animal laboratory at Zhongnan Hospital of Wuhan University. The mice were acclimatized to the laboratory environment for 1 week before experiments.

### Experimental protocol

Mice were randomly divided into 2 groups: control group and Surgery/Anesthesia group. As demonstrated in Figure 1, the mice in the Surgery/Anesthesia group were subjected to a simple laparotomy under isoflurane anesthesia, while the mice in the control group were placed in their home cages and exposed to 100% oxygen for two hours without surgical treatments. The mice had multiple behavioral tests at 24 hours before (baseline) and 6, 9, and 24 hours after the Surgery/Anesthesia. Within each group, separate cohorts were subjected to assessments at each time point (n = 9 per cohort). Mice were anesthetized and decapitated immediately and at 6, 9, and 24 hours after the Surgery/Anesthesia, and hippocampal and prefrontal cortex tissues were harvested for analysis of DISC1, Drp1, Mfn2, ROS, SOD, MMP, ATP, PSD-95, and BDNF levels (n = 6 per cohort). Mice were anesthetized and transcardially perfused with ice-cold phosphate-buffered saline (PBS) followed by paraformaldehyde and glutaraldehyde. Then, hippocampal and prefrontal cortex tissues were collected and stored in the same fixative for electron microscopy analysis immediately and at 24 hours after the Surgery/Anesthesia (n = 3 per cohort).

### Surgical model

A simple laparotomy was performed under isoflurane anesthesia using the methods described in our previous studies [32]. Specifically, anesthesia was induced and maintained using 1.4% isoflurane in 100% oxygen in a transparent acrylic chamber. Fifteen minutes after the induction, the mouse was moved out of the chamber, and isoflurane anesthesia was maintained via a cone device. A 16-gauge needle was inserted into the cone near the nose of the mouse to monitor the concentration of isoflurane. A longitudinal midline incision was made from the xiphoid to the 0.5 centimeter proximal pubic symphysis through the skin, abdominal muscles, and peritoneum. Then, the incision was sutured layer by layer using 5–0 Vicryl thread. The procedure for each mouse lasted about ten minutes, and the mouse was then put back into the anesthesia chamber for up to 2 hours to receive the rest of the anesthesia consisting of 1.4% isoflurane in 100% oxygen. A heat pad was used to keep the mouse body temperature between 36 °C and 37 °C during the surgery. In our preliminary experiments, a mouse-tail blood pressure cuff (Softron BP-2010A; Softron Beijing Biotechnology Co. Ltd., Beijing, China) was used to measure blood pressure, and blood gas and blood glucose levels were determined using a blood gas analyzer (i-STAT; Abbott Point of Care Inc., Princeton, NJ, USA). We found that Surgery/Anesthesia does not cause significant cardio-respiratory and/or metabolic disturbances in C57BL/6J mice (see Additional file 1). To treat the pain associated with the incision, 2% lidocaine solution was applied locally before the incision was made, and EMLA cream (2.5% lidocaine and 2.5% prilocaine) was applied to the incision wound at the end of the procedure and then every eight hours for a day.

### Behavioral tests

The behavioral changes were detected using multiple behavioral tests in the following order—buried food test, open field test, and finally Y maze test—at 24 hours before (baseline) the Surgery/Anesthesia and at 6, 9, and 24 hours postoperatively as described in our previous studies [32]. In all tests, each apparatus was cleaned with 75% ethanol after each mouse to remove odors.

### Buried food test

The buried food test was performed as described in previous studies [71, 72] with modifications. Specifically, two days before the test, each mouse was given two pieces of sweetened cereal. On all test days, we habituated the mice for one hour prior to the test by placing the home cage with mice in the testing room. The test cage was prepared with clean bedding (3 centimeters high). One sweetened cereal pellet was buried 0.5 centimeter below the surface of bedding so that it was not visible. The location of the food pellet was changed every time in a random fashion. The mouse was placed in the center of the test cage, and the latency of that mouse to eat the food was measured. Latency was defined as the time from when the mouse was placed in the test cage until when the mouse uncovered the food pellet and grasped it in the forepaws and/or teeth. Mice were allowed to eat the pellet they found and were then returned to their home cage. The observation time was 5 minutes. If the mouse could not find the pellet within 5 minutes, the testing session ended, and the latency was defined as 300 seconds for that mouse.

### Open field test

The open field test was performed as described in previous studies with modifications [73, 74]. Specifically, each mouse was placed in the center of an open field chamber (40 × 40 × 40 centimeters) under dim light and was allowed to move freely for 5 minutes. The movement parameters of the mouse were monitored and analyzed via a video camera connected to the Any-Maze animal tracking system software (Xinruan Information Technology Co. Ltd., Shanghai, China). The total distance moved (millimeters), the freezing time (seconds), the time (seconds) spent in the center of the open field, and the latency (the time in seconds for the mice to reach the location at the first attempt) to the center of the open field were recorded and analyzed.

### Y maze test

The Y maze test was performed as described in the previous studies with modifications [75, 76]. Specifically, the Y maze was placed in a quiet and illuminated room. Each maze consisted of three arms (8 × 30 × 15 centimeters, width × length × height) with an angle of 120 degrees between each arm. The three arms included the start arm, in which the mouse starts to explore (always open), the novel arm (blocked at the first trial but opened at the second trial), and the other arm (always open). The start arm and other arm were designated randomly to avoid spatial memory error. The Y maze test consisted of two trials separated by an inter-trial interval (ITI). The first trial (training) was 10 minutes in duration and allowed the mouse to explore two arms (the start arm and other arm) of the maze, with the novel arm being blocked. After a 2 hour (for the studies of 6 and 24 hours after the Surgery/Anesthesia) or 4 hour (for the study of 9 hours after the Surgery/Anesthesia) ITI, the second trial (retention) was conducted. For the second trial, the mouse was placed back in the maze in the same start arm with free access to all three arms for 5 minutes. A video camera, which was linked to the Any-Maze animal tracking system software, was installed 60 centimeters above the chamber to monitor and analyze the number of entries and the time spent in each arm. The time spent in and entries into the novel arms indicated the spatial recognition memory (learned behavior).

### Brain tissue harvest, lysis, and protein quantification

Mice were anesthetized and euthanized immediately and at 6, 9, and 24 hours after the Surgery/Anesthesia, and hippocampal and prefrontal cortex tissues were harvested, with a portion of the tissues dissected from one side for western blot analysis of DISC1, PSD-95, and BDNF levels. Briefly, the harvested brain tissues were homogenized on ice using immunoprecipitation buffer (10 mM Tris-HCl, pH 7.4, 150 mM NaCl, 2 mM EDTA, 0.5% Nonidet P-40) with protease inhibitors (1 mg/ml aprotinin, 1 mg/ml leupeptin, 1 mg/ml pepstatin A). The lysates were collected and centrifuged at 13,000 g for 5 minutes at 4 °C. The protein concentrations were determined using the bicinchoninic acid protein assay kit (Aspen Biotechnology, Wuhan, China) according to the manufacturer’s instructions.

### Isolation of mitochondria from the hippocampus and prefrontal cortex

Intact mitochondria were isolated from fresh hippocampus and prefrontal cortex tissues using a tissue mitochondria isolation kit (Beyotime Biotechnology, Shanghai, China). In brief, after brain tissues were homogenized in ice-cold MSH buffer (10 mM HEPES, pH 7.5, 200 mM mannitol, 70 mM sucrose, 1.0 mM EGTA, and 2.0 mg/ml serum albumin), and the homogenate was centrifuged at 1,000 g for 10 min at 4 °C. The collected supernatant was then centrifuged at 3,500 g for 10 min at 4 °C to obtain the mitochondrial pellet; then, the collected supernatant was centrifuged at 12,000 g for 10 min at 4 °C to obtain pure cytosol fractions.

### Western blot analysis

Equal amounts of the sample (40 μg of protein) were separated on gradient sodium dodecyl sulphate polyacrylamide gels and transferred onto a polyvinylidene fluoride membrane (Millipore, MA, USA), which was then blocked with 5% skimmed milk solution. Afterward, the membranes were incubated with primary antibodies overnight at 4 °C. The primary antibodies used in this study were rabbit anti-DISC1 (1:2,000; Abcam, Cambridge, UK), rabbit anti-Drp1 (1:1,500; Abcam), rabbit anti-Mfn2 (1:1,000; Abcam), rabbit anti-PSD-95 (1:1,000; Abcam), rabbit anti-BDNF (1:2,000; Abcam), rabbit anti-β-actin (1:10,000; Beijing TDY Biotech Co. Ltd., Beijing, China) and rabbit anti-porin (1:1,000; Abcam). After three washes with TBST buffer, the membranes were incubated with the appropriate secondary antibody, HRP-goat anti rabbit (1:10,000; Aspen Biotechnology). Immunoreactive bands were visualized using an enhanced chemiluminescence kit (Aspen Biotechnology) and X-ray films. Image analysis was completed using AlphaEaseFC software (Alpha Innotech, CA, USA). The ratio between the protein of interest and β-actin (or porin) levels were first obtained and then expressed as a percent change from a control density.

### Assay for the levels of SOD, ROS, and ATP

The hippocampal and prefrontal cortex tissues were washed in ice-cold isotonic saline solution, homogenized on ice, and then centrifuged at 12,000 rpm for 20 min at 4 °C to obtain the supernatant. All samples were stored at –80 °C. The levels of SOD, ROS, and ATP were determined using the SOD Assay Kit (Nanjing Jiancheng Bioengineering Institute, Nanjing, China), ROS Assay Kit (Beyotime Biotechnology), and ATP Assay Kit (Nanjing Jiancheng Bioengineering Institute), respectively, according to the manufacturer’s instructions. The concentrations of the protein were quantified with reference to the standard curve.

### Measurement of MMP

Fresh hippocampal and prefrontal cortex tissues were used for measurement of MMP. Changes in MMP was measured using a JC-1 mitochondrial membrane potential assay kit (Beyotime Biotechnology) according to the manufacturer’s instructions. Flow cytometry was performed using a digital flow cytometer (BD FACSAria; BD Biosciences, NJ, USA) at an excitation wavelength of 488 nm.

### Transmission electron microscopy analysis

Mice were anesthetized and transcardially perfused with ice-cold PBS followed by 4% paraformaldehyde; then, hippocampal tissues were collected and stored in 2.5% glutaraldehyde for electron microscopy analysis immediately after the Surgery/Anesthesia and at 24 hours postoperatively. Our protocol for morphometric analyses of mitochondria using electron microscopy has been described previously [40, 77, 78]. Briefly, the tissues were fixed in 2% osmium tetroxide, stained with 4% uranyl acetate, embedded in aclar sheets using epon–araldite resins, and sectioned (50–75 μm thick) using a microslicer (Leica Biosystems, Shanghai, China). The slices were then dehydrated in graded aqueous solutions of 40 to 96% ethanol (10 min each) and finally in 100% acetone (three washes, 10 min each) before being embedded in a capsule of pure epoxy resin. Ultrathin sections were placed on grids and examined using an electron microscope (Olympus, Tokyo, Japan).

### Statistical analysis

The statistical analysis was performed using SPSS 19.0 statistical software (IBM Co.; Armonk, NY, USA) or GraphPad Prism 6 software for Windows (GraphPad Software Inc.; La Jolla, CA, USA). The normality of the data was analyzed using the Shapiro-Wilk test, and the data was found to be normally distributed. The quantitative data are expressed as the mean±standard error of the mean (SEM), with the error bars indicating the SEM. Different groups were compared using the Student’s *t*-test. A value of *p*<0.05 was considered statistically significant.

## Supplementary Material

Supplementary Figure 1
