# Supplementary material for: Surgery/Anesthesia disturbs mitochondrial fission/fusion dynamics in the brain of aged mice with postoperative delirium
Source: Aging (Albany NY). 2020 Jan 12;12(1):844–65. doi: 10.18632/aging.102659 (PMC6977661; doi:10.18632/aging.102659)
Supplement: Supplementary Figure 1 [file aging-12-102659-s001..pdf]

SUPPLEMENTARY FIGURE

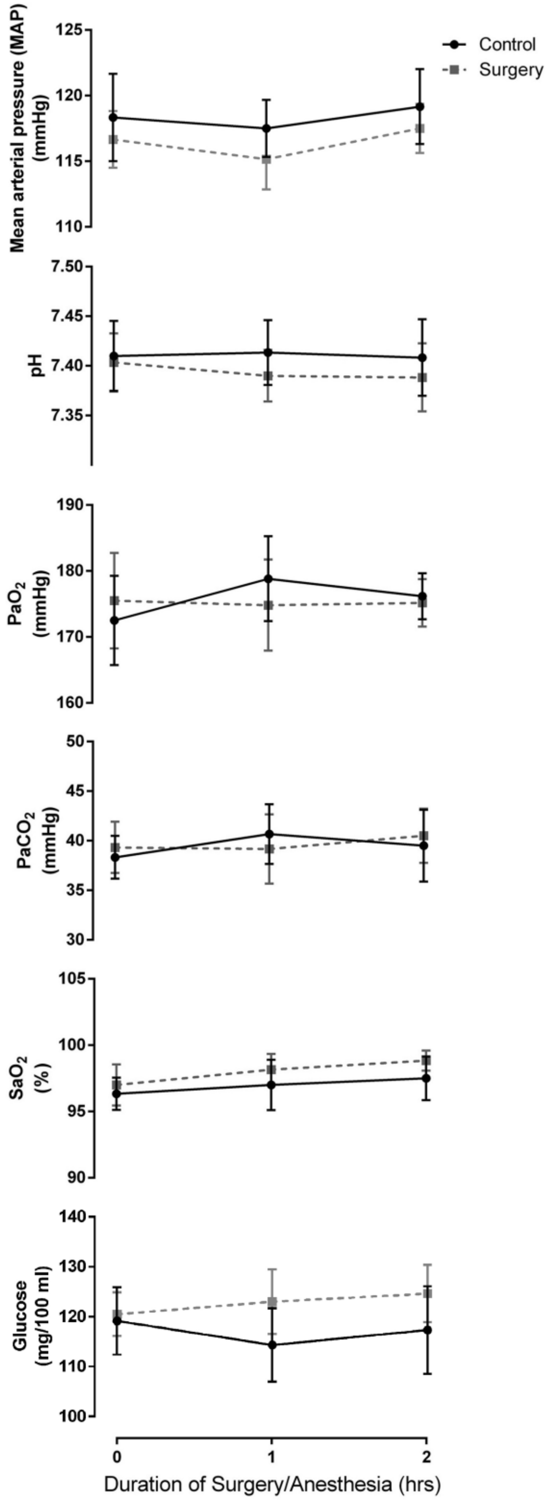

Supplementary Figure 1. The physiological changes in the mouse model of surgery. Surgery/ Anesthesia does not cause significant cardio-respiratory and/or metabolic disturbances in C57BL/6J mice. Analysis of the intraoperative blood pressure and arterial blood gases every hour during Surgery/Anesthesia revealed no significant differences in any of the measured parameters compared to the control condition. The data are plotted as the mean ± standard error of the mean for each group (n=6).
